# Solution NMR assignment of the ARC4 domain of human tankyrase 2

**DOI:** 10.1007/s12104-019-09887-w

**Published:** 2019-03-07

**Authors:** Mariola Zaleska, Katie Pollock, Ian Collins, Sebastian Guettler, Mark Pfuhl

**Affiliations:** 1 Divisions of Structural Biology & Cancer Biology, The Institute of Cancer Research (ICR), London, SW7 3RP UK; 2 Division of Cancer Therapeutics, The Institute of Cancer Research (ICR), London, SW7 3RP UK; 3 School of Cardiovascular Medicine and Sciences and Randall Centre, King’s College London, Guy’s Campus, London, SE1 1UL UK

**Keywords:** ADP-ribosylation, Ubiquitylation, Ankyrin repeats, Signaling

## Abstract

Tankyrases are poly(ADP-ribose)polymerases (PARPs) which recognize their substrates via their ankyrin repeat cluster (ARC) domains. The human tankyrases (TNKS/TNKS2) contain five ARCs in their extensive N-terminal region; of these, four bind peptides present within tankyrase interactors and substrates. These short, linear segments, known as tankyrase-binding motifs (TBMs), contain some highly conserved features: an arginine at position 1, which occupies a predominantly acidic binding site, and a glycine at position 6 that is sandwiched between two aromatic side chains on the surface of the ARC domain. Tankyrases are involved in a multitude of biological functions, amongst them Wnt/β-catenin signaling, the maintenance of telomeres, glucose metabolism, spindle formation, the DNA damage response and Hippo signaling. As many of these are relevant to human disease, tankyrase is an important target candidate for drug development. With the emergence of non-catalytic (scaffolding) functions of tankyrase, it seems attractive to interfere with ARC function rather than the enzymatic activity of tankyrase. To study the mechanism of ARC-dependent recruitment of tankyrase binders and enable protein-observed NMR screening methods, we have as the first step obtained a full backbone and partial side chain assignment of TNKS2 ARC4. The assignment highlights some of the unusual structural features of the ARC domain.

## Biological context

The tankyrases (TNKS/ARTD5, TNKS2/ARTD6) are poly(ADP-ribose)polymerases (PARPs) and as such catalyse the processive modification of protein substrates with poly(ADP-ribose) (PAR) chains, thereby consuming their co-substrate NAD^+^ (Haikarainen et al. 2014). PARPs are part of a larger family of Diphtheria-toxin-like ADP-ribosyltransferases (ARTDs), which share variants of a conserved catalytic domain that either modifies substrates with mono-ADP-ribose or PAR, or lacks detectable catalytic activity (Hottiger et al. 2010; Vyas et al. 2014). Different ARTD family members are distinguished by unique combinations of accessory domains, which confer functional diversity (Hottiger et al. 2010). In the case of the tankyrases, these are an extensive N-terminal region comprising five consecutive ankyrin repeat clusters (ARCs), either flexibly or rigidly linked, and responsible for substrate recruitment (Seimiya et al. 2004; Guettler et al. 2011; Eisemann et al. 2016). The ARCs are followed by a polymerizing sterile alpha motif (SAM) domain (De Rycker and Price 2004; Mariotti et al. 2016; Riccio et al. 2016) that precedes the PARP domain (Lehtiö et al. 2008). ARCs and the SAM domain direct tankyrase to regulators of a wide range of biological processes, among which Wnt/β-catenin signaling (Huang et al. 2009; Mariotti et al. 2016, 2017; Yang et al. 2016), the maintenance and mitotic resolution of telomeres (Smith et al. 1998; Smith and de Lange 2000; Dynek and Smith 2004) and glucose metabolism (Chi and Lodish 2000; Yeh et al. 2007; Zhong et al. 2016) are some of the best-studied. Additional roles of tankyrase include the regulation of mitotic spindle formation (Chang et al. 2005, 2009), Hippo signaling (Wang et al. 2015; Troilo et al. 2016; Jia et al. 2017) and emerging functions in the DNA damage response (Nagy et al. 2016), cell migration (Lupo et al. 2016), and Notch signaling (Bhardwaj et al. 2017), to name a few. Proteomics studies and in-silico predictions of tankyrase binders illustrate the diverse cellular functions of tankyrase (Guettler et al. 2011; Li et al. 2017; Bhardwaj et al. 2017).

Of the five ARCs, four (ARCs 1, 2, 4 and 5) are known to bind substrates featuring a degenerate six- to eight-amino-acid peptide motif known as the tankyrase-binding motif (TBM); ARC3 shows no detectable substrate binding (Sbodio and Chi 2002; Seimiya et al. 2004; Guettler et al. 2011). Studies reported in 2011 and 2012 first revealed the structural basis of substrate recruitment by tankyrase (Guettler et al. 2011; Morrone et al. 2012). To date, numerous additional crystal structures of tankyrase ARCs from both TNKS and TNKS2, typically complexed with TBM peptides, are available (Li et al. 2016; Eisemann et al. 2016; Xu et al. 2017a, b; DaRosa et al. 2018).

The TBM binding site in substrate-binding ARCs is highly conserved (Guettler et al. 2011). It features a sub-pocket, the so-called ‘arginine cradle’, that engages an essential arginine residue at TBM position 1. A second key binding determinant is a channel lined by two aromatic amino acids which accommodates an essential glycine residue at TBM position 6, forming an ‘aromatic glycine sandwich’ (Guettler et al. 2011). Their strict requirement suggests that the arginine and glycine residues represent critical docking points in substrate recruitment. Additional contacts are provided by residues at TBM positions 4 and 5, which interact with a ‘central patch’ in the peptide-binding pocket, and at TBM position 8, where an acidic residue can confer increased binding affinity through a salt bridge (Guettler et al. 2011). The eight amino acids of the TBM do not need to be contiguous: the essential arginine can be N-terminally displaced to varying extent, and structural plasticity or looping of such TBM peptides enables both the arginine and a hydrophobic side chain at position 4 to dock as in “canonical” TBM peptides (Morrone et al. 2012; DaRosa et al. 2018). Moreover, there is evidence for tankyrase targets devoid of detectable TBM sequences (Li et al. 2017; Bhardwaj et al. 2017). These binders may either indirectly interact with tankyrase or be recruited through direct binding by alternative, hitherto unknown binding mechanisms.

Tankyrases are being explored as potential therapeutic targets in conditions such as cancer, neurodegeneration, fibrosis and diabetes (Riffell et al. 2012; Haikarainen et al. 2014; Zhong et al. 2016; Mariotti et al. 2017). These efforts led to the development of a wide range of tankyrase catalytic inhibitors (Haikarainen et al. 2014; Mariotti et al. 2017). The response of tankyrase to catalytic inhibition, however, is complex. It not only leads to the loss of tankyrase’s enzymatic PARP activity but typically also to tankyrase accumulation and that of many of its substrates (Huang et al. 2009; Zhang et al. 2011; Bhardwaj et al. 2017) and potentially to increased tankyrase polymerization (De Rycker and Price 2004). The accumulation of tankyrase and its substrates upon tankyrase catalytic inhibition is a consequence of an attenuated PAR-dependent ubiquitination pathway (Zhang et al. 2011; DaRosa et al. 2015). Moreover, some activities of tankyrase may be mediated by non-catalytic (scaffolding) mechanisms: at least elevated tankyrase levels can drive Wnt/β-catenin signalling independently of PARylation, requiring only the substrate-binding ARCs and the polymerizing SAM domain (Mariotti et al. 2016). Consequently, targeting tankyrase’s accessory domains provides an attractive alternative means to inhibit tankyrase function. This approach would block both tankyrase-dependent scaffolding and substrate PARylation. As proof of concept, a recent study demonstrates that an affinity-optimised TBM (Guettler et al. 2011), when stabilised by cyclisation and fused to a facilitator of cell permeability, can inhibit Wnt/β-catenin signalling in cells (Xu et al. 2017b).

Here, we report the assignment of ARC4 of human TNKS2, which will enable a more elaborate characterisation of substrate recruitment by tankyrase ARCs and the development of substrate binding antagonists.

## Methods and experiments

The expression construct for TNKS2 ARC4 (NM_025235), comprising residues 488–649 cloned into vector pETM-30, was described previously (Guettler et al. 2011). To produce ^15^N-labelled or ^15^N–^13^C-labelled protein, *E. coli* (BL21-CodonPlus(DE3)-RIL) cells were grown in M9 minimal media (Laboratory Support Services, ICR) supplemented with ^15^NH_4_Cl or ^15^NH_4_Cl (at 1 g/L M9) and ^13^C d-glucose (Cambridge Isotope Laboratories) (at 6 g/L M9), respectively, following the protocol of Marley and colleagues (Marley et al. 2001). Briefly, freshly transformed bacteria were grown at 37 °C in 4 L of standard LB media (Laboratory Support Services, ICR) containing kanamycin (50 µg/mL) and chloramphenicol (34 µg/mL) until they reached an OD_600_ of 0.7. At this point, cells were transferred into M9 minimal media containing appropriate isotope(s) by collecting cells by centrifugation and resuspending them in 1 L of the final M9 minimal media. The culture was incubated at 37 °C for 1 h to allow the cells to recover, and protein expression was induced by addition of 0.5 mM IPTG. Protein expression was carried out at 18 °C for 16 h. Cells were next harvested by centrifugation, and the pellet was stored at − 80 °C until purification following the previously described method (Guettler et al. 2011; Pollock et al. 2017).

NMR samples were prepared in a buffer of 20 mM sodium phosphate pH 7.0, 100 mM sodium chloride and 1 mM TCEP with a protein concentration of 1 mM. Backbone and partial sidechain assignments of the domain were obtained from a combination of 3D HNCACB, (H)C(CCO)NH, HNCO, HN(CA)CO and ^15^N resolved 3D NOESY-HSQC experiments recorded at 700 and 800 MHz on Bruker Avance spectrometers at 20 °C. Spectra were processed with Topspin 3.1 (Bruker), and all assignments were performed with CCPN analysis 2.4 (Vranken et al. 2005).

### Assignments and data deposition

**Fig. 1:**
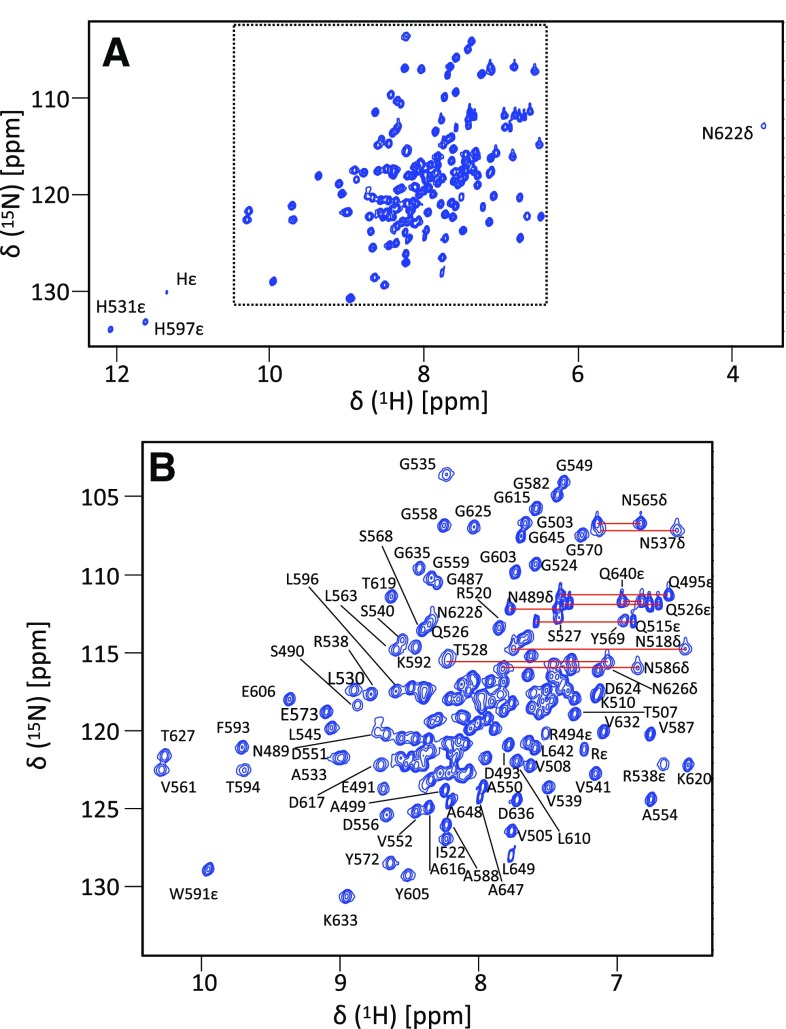
^1^H-^15^N HSQC spectrum of 1 mM uniformly ^15^N/^13^C labelled ARC4 recorded at a temperature of 293 K and a field of 700 MHz. Note that the sidechain resonances of histidines and arginines are folded from their original position in the ^15^N dimension. **a** Overview spectrum. **b** Majority of the backbone resonances in the spectrum (indicated by box in **a**). Well resolved peaks have been labelled with their assignments; pairs of peaks for sidechain NH_2_ groups are connected by red lines

The TNKS2 ARC4 domain construct comprises 165 amino acids (residues 488–649 plus 3 additional N-terminal amino acids, GAM, resulting from the cloning method) and despite its substantial molecular weight of 17.8 kDa gives excellent NMR spectra (see Fig. 1). It was possible to find assignments for 164 residues with only the N-terminal glycine missing completely. Of the assigned residues, backbone amide peaks were missing for only two of the non-proline residues (R525 and V584). Out of a total of 165 backbone nitrogens, 165 α carbons, 151 β carbons, 140 γ carbons, 99 δ carbons, 31 ε carbons and 165 backbone carbonyls, a total of 156 (94.5%), 163 (98.7%), 148 (98.0%), 68 (48.6%), 42 (42.4%) and 11 (35.4%), respectively, could be assigned. The assignment has been deposited with the BMRB, accession code 27747.

## Comparison to X-ray structure

**Fig. 2:**
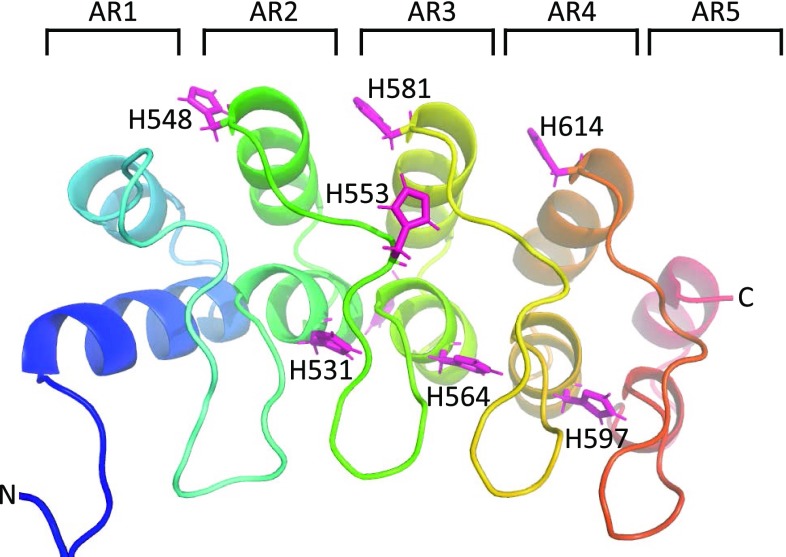
Structure of ARC4 (PDB code 3TWQ) showing all histidine side chains as sticks in pink while the main backbone is shown in cartoon style coloured by sequence from N-terminus (blue, left) to C-terminus (red, right). Ankyrin repeats (ARs) are numbered AR1 to AR5. The positions of the histidines occupying key positions in the three central ARs are clearly visible with H548, H581 and H614 pointing towards the solvent (top) while H531, H564 and H597 (bottom) are covered by the β-hairpins linking ARs. H553 (front) and H571 (back, not labelled) are not part of the conserved AR pattern and exposed to solvent

The NMR spectra of ARC4 contain a number of unusual features, most prominently the appearance of 3 peaks for histidine sidechain Nε2/Hε2 groups, two of which could be assigned. Such resonances are usually exchange-broadened beyond detection. ARC4, however, makes a rather unusual use of histidines as part of the conserved ankyrin repeat (AR) infrastructure, with regular occurrences at the N-terminal end of the first helix and the C-terminal end of the second helix of each AR. Thus, they appear on opposite sides of the protein in the central three ARs (see Fig. 2). Those at the C-termini of the second helix point into solution whereas those at the N-termini of the first are covered by the long loop/beta hairpins which connect adjacent ARs. The latter are part of a highly conserved TPLH sequence motif (Mosavi et al. 2002). As part of this motif, the histidine Hε2 acts as an H-bond donor to the backbone carbonyl group of the residue preceding the TPLH motif in the following repeat (Preimesberger et al. 2015). Our observation of three histidine sidechain Nε2/Hε2 groups is therefore in good agreement with the structure. Curiously, the C_α_ secondary shifts of two of these three protected histidines (H531 with almost 9 ppm, H564 with almost 10 ppm; H597 with 5.5 ppm is less extreme but still substantial) are well outside the [− 5.0, + 5.0] bracket of all the other residues (see Fig. 2). This pattern is less apparent for the C_β_ and C’ secondary shifts. However, it is repeated, albeit with a shift of − 3 in the sequence, for the H_α_ secondary chemical shifts: residues T528, V561 and T594 have values of around + 1.4, much larger than all the other values, which are well within the [− 0.8, + 0.8 ppm] bracket. It is not completely clear what causes the unusual chemical shifts. The fact that these instances of unusual secondary chemical shifts occur in precisely repeated structural units in AR–AR boundaries suggests that the most unusual backbone conformation, in combination with the hydrogen bonds from the histidine side chains to the backbone amides i-3 (Preimesberger et al. 2015), are likely to be the causative factor.

Another unusual feature is the sidechain amide group of N622, which has proton resonances at 8.33 and 3.59 ppm. The extreme shift of one of the amides can be explained by its position extremely close to the aromatic ring of F593, with which it is very likely to form a π–hydrogen bond.

Finally, we can observe three arginine sidechain Nε, which is also not common at pH values around 7. Two of these could be assigned to R538 and R494. R494 is very likely involved in simultaneous salt bridges with the neighbouring E491 and E523 (shortest distances from R494Hη11–E491 Oε2: 2.0 Å; R494Hε–E523 Oε2: 3.0 Å). The Hε of R538 is likely to make a hydrogen bond to the backbone carbonyl oxygen of K501 (distance R538 Hε-K501 O’: 2.0 Å).

### Secondary structure

**Fig. 3:**
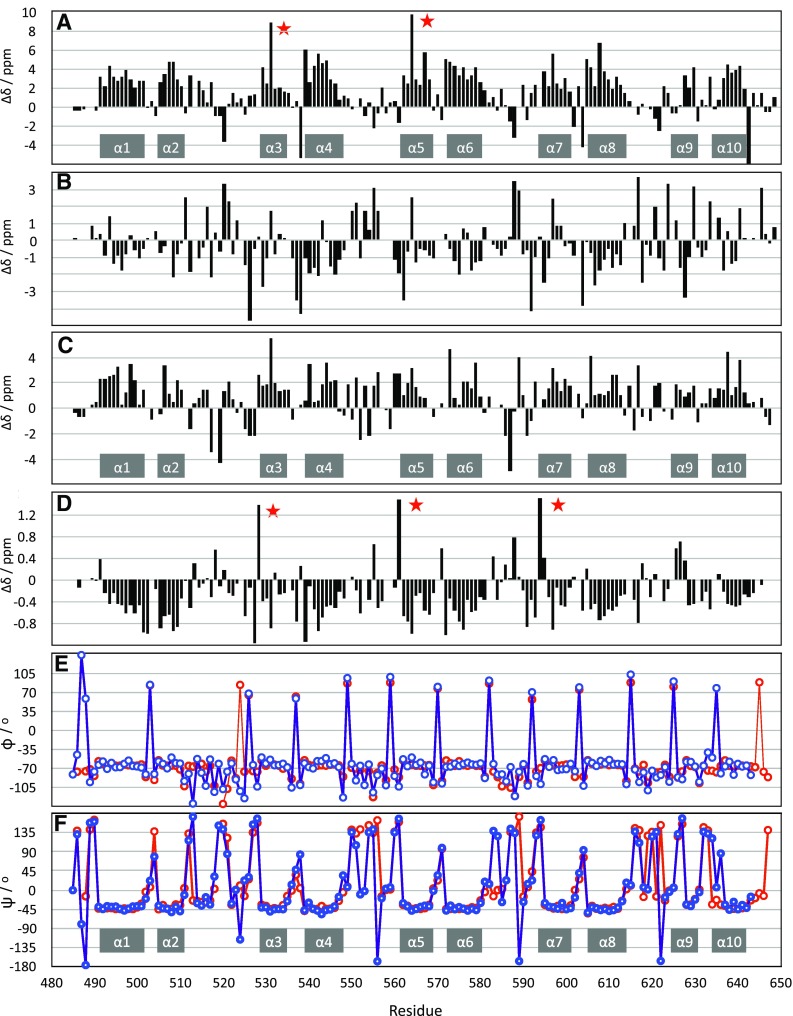
Secondary chemical shifts (Wishart and Sykes 1994) and backbone dihedral angles predicted from Dangle and extracted from the crystal structure of ARC4 (PDB code 3TWQ) (Guettler et al. 2011) All values were calculated using CCPN analysis version 2.4, and figures were generated in Apple Numbers, inkscape and keynote. **a** Cα, **b** Cβ, **c** C’, **d** Hα, **e** phi, **f** psi. Dihedral angles from Dangle are shown in red, those from the crystal structure in blue. Positions of secondary structure elements based on the chemical shift analysis are indicated as grey bars. Positions of the protected histidines with chemical shift outliers are indicated by red stars

We analysed the secondary structure of ARC4, based on the backbone chemical shifts, and compared it to the crystal structure (Guettler et al. 2011) (PDB code 3TWQ). In the first instance, we qualitatively compared the positions of the helices; we next quantitatively compared the backbone dihedral angles predicted by Dangle (Cheung et al. 2010) to those extracted from the crystal structure (see Fig. 3). The positions of the helices are generally in good agreement with the individual secondary chemical shifts, especially the C_α_ and H_α_ values, while C’ and C_β_ provide a less clear correlation. The only exception is helix 9 where some values for H_α_ deviate somewhat. Even more intriguing is the result of the Dangle analysis. We can see an excellent agreement of the values with those from the crystal structure. Most importantly, the sharp changes of phi and psi between the helices are precisely matched for most residues. (Note the apparently huge difference in psi prior to the first helix of each AR (H1, H3, H5, H7, H9), which is actually very small due to the circular periodicity of the dihedral value; i.e., a value of + 175° is actually very close to − 175°.) The only deviations are seen prior to helix 3, near to H531 and at the C-terminus. The region around the former folds in an unusual way and involves rare interactions which cause unusual chemical shifts as outlined above. At the latter, the conformation is less well defined and likely to differ between solution and crystal. We can therefore conclude that for a protein with a low level of conformational dynamics, and thus a very narrow distribution of conformations in solution and in the crystal, we can extract very precise backbone dihedral angle constraints.
